# Lung function decline in subjects with and without COPD in a population-based cohort in Latin-America

**DOI:** 10.1371/journal.pone.0177032

**Published:** 2017-05-04

**Authors:** Rogelio Pérez-Padilla, Rosario Fernandez-Plata, Maria Montes de Oca, Maria Victorina Lopez-Varela, Jose R. Jardim, Adriana Muiño, Gonzalo Valdivia, Ana Maria B. Menezes, On behalf of the PLATINO group

**Affiliations:** 1 National Institute of Respiratory Diseases, Mexico City, Mexico; 2 Pulmonary Division, Hospital Universitario de Caracas, Universidad Central de Venezuela, Caracas, Venezuela; 3 University of the Republic, Faculty of Medicine, Montevideo, Uruguay; 4 School of Medicine, Federal University of Sao Paulo, Pelotas, Brazil; 5 Facultad de Medicina, Pontificia Universidad Católica de Chile, Santiago, Chile; 6 Post-graduate Program in Epidemiology, Federal University of Pelotas, Pelotas, Brazil; Lee Kong Chian School of Medicine, SINGAPORE

## Abstract

**Background:**

Lung-function decline is one of the possible mechanisms leading to Chronic Obstructive Pulmonary Disease (COPD).

**Methods:**

We analyzed data obtained from two population-based surveys of adults (n = 2026) conducted in the same individuals 5–9 years (y) after their baseline examination in three Latin-American cities. Post BronchoDilator (postBD) FEV_1_ decline in mL/y, as %predicted/y (%P/y) and % of baseline/y (%B/y) was calculated and the influence of age, gender, BMI, baseline lung function, BD response, exacerbations rate evaluated using multivariate models.

**Results:**

Expressed in ml/y, the mean annual postBD FEV_1_ decline was 27 mL (0.22%P, 1.32%B) in patients with baseline COPD and 36 (0.14%P, 1.36%B) in those without. Faster decline (in mL/y) was associated with higher baseline lung function, with significant response to bronchodilators, older age and smoking at baseline, also in women with chronic cough and phlegm, or ≥2 respiratory exacerbations in the previous year, and in men with asthma.

**Conclusions:**

Lung function decline in a population-based cohort did not differ in obstructed and non-obstructed individuals, it was proportional to baseline FEV_1_, and was higher in smokers, elderly, and women with respiratory symptoms.

## Introduction

Chronic Obstructive Pulmonary Diseases (COPD) is a leading cause of death worldwide. Classically, COPD has been considered a consequence of rapid lung-function decline during adult life, but more recently, it has also been considered as the consequence of poor lung development either before or after birth[1,2].

There is a great interest in identifying COPD subgroups, the so-called COPD phenotypes which are defined as ‘‘a single or a combination of disease attributes that describe differences between individuals with COPD as they relate to clinically meaningful outcomes (symptoms, exacerbations, response to therapy, rate of disease progression or death)” [3] In the (PLATINO) study population, we have described the main characteristics of several phenotypes associated with COPD[4–6]. Recently we reported longitudinal mortality rates according to COPD status and lung function measured at baseline in population-based samples of adults living in three Latin American cities, with follow-up periods ranging from 5–9 years (y) (the PLATINO follow-up study)[7]. To our knowledge, no information is available regarding lung-function decline in Latin America or its determinants in a population-based study; we aimed to assess the decline in the Forced Expiratory Volume in the first second (FEV_1_) in a large sample from Latin America with a high overall response rates and robust, well-established methods.

## Methods

The study protocol was approved by the Ethics Committee on Research, Pontificial Catholic University of Chile School of Medicine, by the Ethics Committee of the Maciel Hospital in Montevideo Uruguay, and by the Ethics Committee on Research of the Federal University of Sao Paulo/Sao Paulo Hospital. Study participants provided signed informed consent. The detailed methods of the PLATINO baseline[8] and follow-up studies[9] are available elsewhere. Between the years 2003 and 2005, population-based surveys were conducted, employing standardized methodology in five large Latin-American metropolitan areas: Sao Paulo (Brazil); Mexico City (Mexico); Montevideo (Uruguay); Santiago (Chile); and Caracas (Venezuela). We successfully interviewed 1,000 subjects aged 40 years or older in Sao Paulo, 1,063 in Mexico City, 943 in Montevideo, 1,208 in Santiago, and 1,357 in Caracas. Spirometry testing was performed for 963 (97.9%) subjects in Sao Paulo, 1,000 (98.3%) in Mexico City, 885 (97.1%) in Montevideo, 1,173 (99.8%) in Santiago, and 1,294 (98.4%) in Caracas[10]. The questionnaires (Spanish and Portuguese) are available at the PLATINO website: http://www.platino-alat.org.

Spirometry in the baseline and follow-up surveys was undertaken using an ultrasonic spirometer (EasyOne; ndd Medical Technologies, Zurich, Switzerland) prior to (pre- BronchoDilator [BD]) and 15 minutes after the administration of 200 μg of Salbutamol (post-BD) according to American Thoracic Society (ATS) criteria of acceptability and reproducibility [11], aiming for >90% test compliance with ATS quality criteria[8].

Follow-up studies were conducted in Montevideo, Santiago, and Sao Paulo 5, 6, and 9 years after the baseline surveys, respectively [9]. Individuals were visited at their homes based on the contact information provided by these persons during the baseline examination.

For the purposes of this work, we defined post-Bronchodilator (postBD) airflow obstruction Chronic Obstructive Pulmonary Disease [COPD] as individuals with a postBD FEV_1_/FVC below the Lower Limit of Normal (LLN)[12–14]–defined as the lower 5^th^ percentile for predicted postBD FEV_1_/FVC based on equations derived from the 5 cities baseline study in a sub-set of healthy and never-smoker subjects[15]. We assessed, in survivors at the second evaluation, the annual decrease in spirometric lung function.

### Statistical analysis

The annual decline in FEV_1_, was estimated by subtracting the second measurement from the first, and subsequently dividing by the number of years (and fractions) between both measurements. The FEV_1_ was analyzed in milliliters, but was also expressed as percentage of predicted (%P) for gender, age, and height[15], as percentage or baseline (%B), and as its natural logarithm[16] both for the preBD and postBD values.

The following potential confounders were explored in a multivariate analysis: age; gender; current smoking at baseline and at final evaluation (dichotomous and as cigarettes/day); lifetime cumulative smoking (pack-years); Body Mass Index BMI (kg/m^2^); years of academic education; self-reported comorbidities (heart disease, hypertension, diabetes, cerebrovascular accident and gastritis or ulcer, separately and as a count of positive comorbidities from 0–5); hour-years of exposure to biomass smoke (average number of years exposed by means of cooking multiplied by the average number of hours per day exposed); years of exposure in an occupation involving dust, smoke, or gases; the presence of chronic bronchitis (cough or phlegm on the majority of days, at least 3 months per year for >2 years, or cough and phlegm for the same time period) and previous Physician diagnosis of asthma or TB; significant response to bronchodilators (a postBD increase in FEV_1_ or FVC of ≥12% and of ≥200mL)[17]; baseline spirometric measurement [15], and the presence of postBD airflow obstruction (COPD). Final multivariate models included all variables showing an association with decline, at a P <0.15.

All statistical analyses were performed using Stata ver 13 statistical software (StataCorp, College Station, TX, USA) and we considered statistical significance as a *P*-value of <0.05.

## Results

Only individuals with valid baseline spriometric data were eligible for follow-up. There were 885 eligible individuals for follow-up in Montevideo, 1173 in Santiago and 963 in Sao Paulo. Information was obtained for 758 (85.6%), 993 (84.7%) and 748 (77.7%) subjects, respectively. A total of 71 deaths in Montevideo, 95 in Santiago and 135 in Sao Paulo were identified. Among the individuals evaluated during follow-up, 2,120 had preBD and 2,026 postBD spirometry tests in both examinations. Follow-up rates for each independent-variable category were around 80% (see flowchart in S1 Appendix) [8,9].

Characteristics of the studied population in the first and second evaluations are depicted in Table 1. Compared with the first examination, individuals in the follow-up exam were older, with less current smoking, and slightly lower lung function.

**Table 1:** Characteristics of the studied population in the first and second evaluation (means± standard deviation [SD]).

Variables	First evaluation	Second evaluation	P-value
Males, (% and 95%CI)	40.9 (39.6–42.3)	40.5 (38.9–42.1)	0.76
Age (years)	57.4±12.1	62.6±11.1	
Height (cm)	160.0±9.7	159.8±9.8	0.99
BMI (kg/m^2^)	28.1±5.7	28.8±5.2	<0.001
FEV_1_ preBD (L)	2.56±0.80	2.41±0.77	<0.001
FVC preBD (L)	3.47±1.02	3.22±0.98	<0.001
FEV_1_ postBD (L)	2.67±0.80	2.50±0.77	<0.001
FVC postBD (L)	3.47±0.98	3.26±0.96	<0.001
FEV_1_/FVC preBD	73.8±9.1	74.9±8.7	<0.001
FEV_1_/FVC postBD	77.1±9.0	76.8±8.5	0.30
Response to bronchodilators, % (95%CI)	9.0 (7.9–10.1)	9.7 (8.4–11.0)	0.013
History of asthma* (95%CI)	15.4 (14.2–16.8)	15.6 (14.1–17.2)	0.86
History of COPD* (95%CI)	4.6 (3.9–5.4)	6.3 (5.3–7.3)	0.01
Current smoker (95%CI)	30.8 (29.1–32.4)	24.3 (22.5–26.1)	<0.001
% exposed to biomass smoke for >6months % (95%CI)	46.2 (44.5–48.0)	27.5 (26.0–29.1)	<0.001
Hour-years in those exposed to biomass smoke (median, IQR)	20 (5, 48)	26 (10, 60)	<0.001 ^Ç^
≥2 exacerbations, last-year % (95%CI)	2.6 (2.0–3.2)	3.3 (2.6–4.1)	0.03
Previous tuberculosis, % (95%CI)	3.4 (2.8–4.1)	2.9 (2.2–3.7)	0.29
Job exposure to dust or smoke, %	47.2 (45.5–49.0)	34.0 (32.3–35.6)	<0.001
Chronic cough or phlegm %	14.5 (13.3–15.8)	17.5 (15.4–18.9)	0.01
Chronic cough and phlegm %(95%CI)	4.2(3.5–4.9)	5.3(4.3–6.3)	0.11
COPD (GOLD stages 1–4), % (95%CI)	17.1 (15.7–18.5)	17.4 (15.8–19.1)	0.76
COPD (GOLD stages 2–4), % (95%CI)	6.5 (5.6–7.4)	6.6 (5.6–7.7)	0.89
COPD (FEV_1_/FVC<LLN), % (95%CI)	9.3 (8.3–10.4)	8.6 (7.4–9.9)	0.37

BMI (Body Mass Index) = weight/ height^2^; SD = Standard Deviation; preBD = before bronchodilator; postBD = after bronchodilator. 95%CI = 95% confidence interval; IQR = interquartile range; GOLD = Global Initiative for Chronic Obstructive Lung Disease. LLN = lower limit of normal; 95%CI = 95% confidence interval. Asthma-COPD overlap = medical diagnosis of asthma (first definition) plus FEV_1_ /FVC<0.7 post-BD or wheezing in the last year plus response to bronchodilator plus FEV_1_/FVC<0.7 in the second definition. Bronchodilator response is the increase in FVC or FEV_1_ of ≥12% and of ≥200mL Chronic bronchitis was cough or phlegm on the majority of days for >3 months in 1 year for >2 consecutive years.

*Self-reported previous physician diagnosis of the condition.

**Student t tests on the equality of means

^Ç^Mann-Whitney U tests, two independent samples. Individuals described are those with spirometric testing at baseline (column one) and follow up (column two, see Methods).

**Fig 1:**
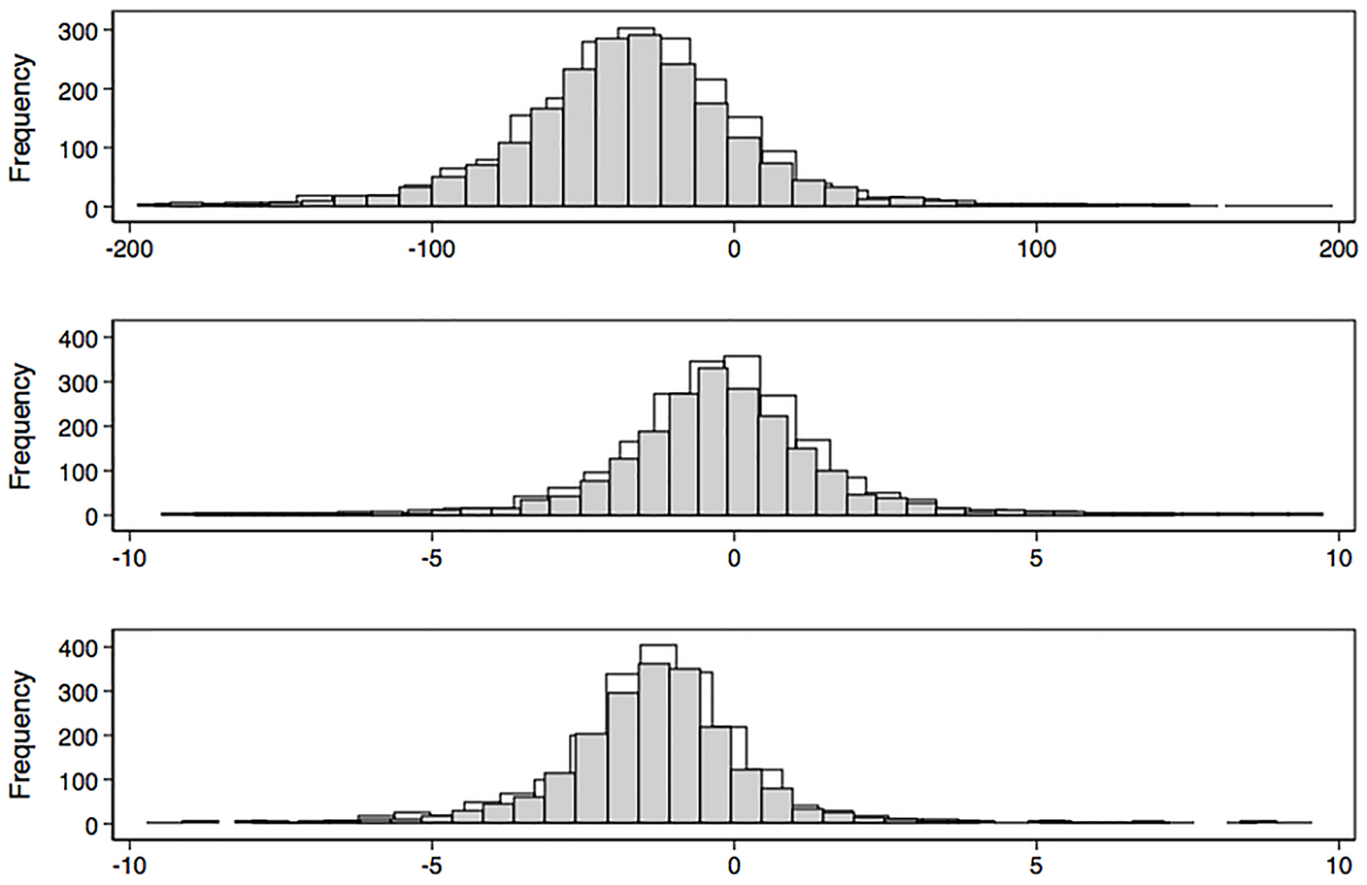
Distribution of pre-bronchodilator (preBD) (gray bars) and post-bronchodilator (postBD) (light bars) Forced Expiratory Volume in the first second (FEV_1_) in mL/y (upper graph); as percentage of predicted (%P, middle graph); and as percentage of baseline (lower graph). Decline varied considerably regardless of in which units is expressed, including individuals with no decline or increase in measurements from baseline.

Decline in spirometric function was heterogeneous (Fig 1), and some individuals did not demonstrate any decline. We observed a second postBD measurement lower than the first, (negative change or decline) in 86% of participants for FEV_1_ expressed in mL, in 58% if function is expressed as %P, and in 86% if expressed as %B. In 5.9% of participants, postBD FEV_1_ increased >20 mL/y during the follow-up (5.6% in women, 6.3% in men, and 9.9% in those with postBD airflow obstruction).

In Table 2, we describe the mean annual decline of FEV_1_, expressed in milliliters, as %P and as %B in women and men. Women declined, on average, for postBD FEV_1_, 31 mL/y (0.17%P/y, 1.33%B/y), whereas men declined on average for postBD FEV_1_ 42 mL/y (0.29%P/y, 1.31%B/y). Decline in men was higher than in women in mL/y but was not statistically different if expressed as %P/y, as %B/y (Table 2), or as **Δ**z scores, or as **Δ**log spirometric values (See Supplementary material, S1–S5 Tables). Decline of postBD measurements >40 mL/y, for FEV_1_, was observed in 34% of women, and in 48%, of men respectively. Similarly, the proportion of individuals with decline of postBD measurements >100 mL/y were higher in men: it was observed in 2.5%, of women, and in 8.5%, of men respectively. Variance of the lung function decline (SD) in the population was significantly higher (P<0.001) for preBD measurements than for posBD measurements, and for men than for women (Table 2).

**Table 2:** Mean annual decline FEV_1_ (SD), with 95% confidence intervals (95%CI) and 5th percentiles (P5).

	Women				Men			
Variables	Mean annual drop (SD)	95%CI, Mean annual decline	5th percentile all	5th percentile healthy	Mean annual drop (SD)	95%CI, Mean annual decline	5th percentile all	5th percentile healthy
preBD FEV_1_ (mL/y)	-29.3 (39.6)	-31.4, -27.1	-88	-100	-40.5 (49.1)	-43.8,-37.2	-125	-149
posBD FEV_1_ (mL/y)	-31.4 (34.8)	-33.5, -29.3	-81	-91	-41.7 (47.7)	-44.9,-38.4	-121	-126
preBD FEV_1_ (%P/y)	-0.14 (1.9)	-0.24, -0.04	-2.9	-3.6	-0.30 (1.7)	-0.41,-0.18	-3.2	-4.2
posBD FEV_1_ (%P/y)	-0.17 (1.7)	-0.27,-0.06	-2.7	-3.1	-0.29 (1.7)	-0.40,-0.18	-3.1	-3.7
preBD FEV_1_ (%B/y)	-1.25 (2.39)	-1.38,-1.12	-4.12	-4.92	-1.28 (2.0)	-1.41,-1.15	-4.31	-4.70
PosBD FEV_1_ (% B/y)	-1.33 (1.61)	-1.43, -1.24	-3.79	-3.91	-1.31 (1.6)	-1.42, -1.20	-4.07	-4.60

%P = expressed as percentage of predicted according to PLATINO reference values. %B = expressed as percentage of baseline. 95%CI = 95% confidence interval of the mean. PreBD = pre bronchodilator test; posBD = post bronchodilator test; SD = standard deviation of the sample. 5th percentile value of the whole cohort (all, 1261 women and 858 men with preBD testing, 1203 and 822 with postBD testing) or from the respiratory healthy (207 women and 104 men with preBD testing and 195, 97 with postBD testing). See the supplemental material for declines of zFEV_1_, FEV1/Height^3^ and LogFEV_1_

Main variables associated with lung function decline are presented in Table 3 and S1–S5 Tables. For postBD values of FEV_1_ (Table 3), decline in men and women was significantly higher in individuals older at baseline, in those with higher baseline lung function, in those with significant response to bronchodilators, and was milder in those with greater height (once baseline lung function was taken into account) (Table 3). When expressed as %P or %B the impact of age was eliminated or reduced but not that of baseline lung function.

**Table 3:** Multivariate regression coefficients (with 95% confidence intervals) for associations with the post bronchodilator Forced Expiratory Volume at one second (FEV_1_, mL/y) decline in the cohort.

	Women				Men			
Variables	FEV_1_ (mL)	95%CI	FEV_1_ (%P)	95%CI	FEV_1_ (mL)	95%CI	FEV_1_ (%P)	95%CI
Baseline PostBD FEV_1_ (mL)	-0.025	-0.031, -0.021	-0.001	-0.001, -0.001	-0.014	-0.020, -0.007		
Age (years)	-0.58	-0.80, -0.36			-0.613	-0.988, -0.238		
Height (cm)	0.65	0.32, 0.98	0.0352	0.0185, 0.0519	0.594	0.068, 1.119	0.013*	-0.0.003, 0.03
BMI (kg/m2)	0.25*	-0.05, 0.54			1.140	0.367, 1.914	0.042	0.01, 0.06
Cigarettes/day	-0.71	-1.0, -0.4	-0.0295	-0.044, -0.015				
Smoking at baseline (yes/no)					-11.85	-18.89, -4.80	-0.33	-0.57, -0.09
TB			-0.661	-1.18, -0.138				
>2 exacerbations last year	-14.8	-24.2, -5.2	-0.559	-1.045, -0.072				
Chronic cough & phlegm	-11.7	-20.8, -2.6	-0.642	-1.11, -0.178				
Response to BD	-11.9	-18.6, -5.2	—0.617	-0.961, -0.274	-25.160	-39.40, -10.92	-0.68	-1.17, -0.19

95%CI = 95% confidence interval of the mean. PreBD = pre bronchodilator test; posBD = post bronchodilator test; %P = expressed as percentage of predicted according to PLATINO reference values. Variability explained by the model (adjusted R2) was 9% in women, and 4.9% in men. Bronchodilator response is the increase in FVC or FEV_1_ of ≥12% and of ≥200mL. Chronic cough and phlegm was cough or phlegm on the majority of days for >3 months in a year for >2 consecutive years. The presence of postBD airflow obstruction (COPD) was not associated with decline in multivariate models. TB = previous tuberculosis. Total number of individuals in the models is 2,120 for preBD and 2,026 por postBD tests.

*All variables included in the models had a P<0.15, but some of the variables in the table did not reach the statistical significance at p<0.05.

The presence of smoking at baseline or the number of cigarettes smoked, were associated with greater decline of FEV_1_ in men and women. Having ≥2 respiratory disease exacerbations in the previous year, and chronic cough and phlegm was associated with higher decline in FEV_1_ in women. Higher BMI predicted a reduced decline of FEV_1_ (Table 3). The presence of a FEV_1_/FVC<LLN (COPD) was not associated with a significant increased decline (Tables 4 and 5), after taking baseline lung function into account.

**Table 4:** Characteristics of subjects with and without Chronic Obstructive Pulmonary Disease (COPD, post-BD FEV_1_/FVC<LLN, mean±standard deviation [SD]) or 95% confidence interval (95%CI) in the whole cohort at baseline.

Variables	NO COPD (N = 2875)	SD (or 95%CI)	COPD (N = 146)	SD (or 95%CI)	P-value*
Males, % (95%CI)	40.6	39.1–41.9	48.6	39.8–57.5	0.06
Age (years)	56.8	11.8	63.1	13.1	<0.001
Height (cm)	160.1	9.7	159.4	10.3	0.43
BMI (kg/m^2^)	28.1	5.7	27.9	5.6	0.75
FEV_1_ preBD (L)	2.60	0.78	1.79	0.76	<0.001
FVC preBD (L)	3.49	1.01	3.09	1.11	<0.001
FEV_1_ postBD (L)	2.71	0.78	1.93	0.76	<0.001
FVC postBD (L)	3.48	0.97	3.32	1.15	<0.001
FEV_1_ /FVC preBD	74.7	8.1	57.3	11.5	<0.001
FEV_1_ /FVC postBD	78.1	7.7	57.5	9.4	<0.001
Deaths (95%CI)	9.1	7.9–11.5	18.5	11.5–25.5	0.001
Bronchodilator response, % (95%CI)	7.4	6.4–8.4	39.7	32.0–47.5	<0.001
History of asthma, % (95%CI)	14.3	13.0–15.7	38.4	30.4–46.3	<0.001
History of COPD, % (95%CI)	4.0	3.2–4.7	17.8	11.1–24.5	<0.001
Current smoker, % (95%CI)	32.9	30.8–35.0	0.000	0.000	<0.001
% exposed to biomass smoke of >6months	54.2	51.8–56.5	64.4	56.7–72.1	0.02
Biomass exposure, (hour-years in exposed) median, IQR	18.6	43.8	36.3	95.5	<0.001
≥1 exacerbations last year, % (95%CI)	2.4	1.8–3.1	8.2	3.8–12.6	<0.001
≥2 exacerbations last year, % (95%CI)	3.1	2.4–3.8	7.4	1.4–13.4	0.04
Chronic cough or phlegm,% (95%CI)	14.2	12.9–15.4	22.6	15.6–29.6	0.01
Chronic cough and phlegm, (95%CI)	4.1	3.4–4.8	8.9	4.2–13.6	0.01
Previous tuberculosis, % (95%CI)	3.1	2.5–3.8	10.3	5.7–14.9	<0.001
Job exposure to dust or smoke, %	47.5	45.4–49.7	58.2	50.4–66.1	0.01

preBD = before bronchodilator use; postBD = after bronchodilator use; COPD is a postBD FEV1/FVC<lower limit of normal according to the PLATINO reference values.

*TTest for independent groups or Fisher exact test for categorical variables; Bronchodilator response is the increase in FVC or FEV_1_ ≥12% &≥200mL. Chronic cough or phlegm on the majority of days for >3 months during a year for >2 years

**Table 5:** Lung function (FEV_1_) decline in participants with and without airflow obstruction (postBD FEV_1_/FVC<LLN), means and standard deviation (SD) or 95%CI.

Variables	NO COPD (N = 2,025)	SD (or 95%CI)	COPD (N = 95)	SD (or 95%CI)	P-value*
Mean decline PreBD (mL/year)	-34.2	43.8	-26.1	48.9	0.08
Mean decline PostBD (mL/y)	-35.9	40.7	-26.8	41.3	0.04
Mean decline PreBD (%P/year)	-0.21	1.8	-0.17	2.1	0.90
Mean decline PostBD (%P/year	-0.22	1.6	-0.14	2.0	0.60
Mean decline PreBD (%B/year)	-1.26	2.2	-1.30	2.8	0.90
Mean decline PostBD (%B/year),	-1.32	1.5	-1.36	2.1	0.80
% with >20 mL/y increase in preBD FEV_1_	5.9	4.9–7.0	7.4	2.0–12.7	0.56
% with >20 mL/y increase in postBD FEV_1_	5.7	4.7–6.7	9.9	3.6–16.1	0.10
% with any preBD FEV_1_ decline	84.5	82.8–86.2	77.9	69.6–86.2	0.08
% with any postBD FEV_1_ decline	86.0	84.5–87.5	80.2	72.4–88.0	0.12
% with any preBD decline as %P	55.8	53.4–58.2	54.7	44.7–64.8	0.84
% with any postBD decline as %P	57.7	55.5–59.9	57.1	46.8–67.5	0.91
% with>40mL/y decline	41.0	38.7–43.3	32.6	23.1–42.2	0.11
% with>40mL/y postBD	42.7	40.4–45.1	38.5	28.6–48.3	0.4
% with preBD decline >3%P/y	4.9	4.0–5.9	8.4	2.7–14.1	0.13
% with postBD decline >3%/y	4.3	3.4–5.2	3.2	0.0–6.7	0.59
% with preBD decline >4%B/y	5.5	4.5–6.5	16.8	9.2–24.5	<0.001
% with postBD decline >4%B/y	4.1	3.2–5.0	7.4	2.0–12.7	0.13

preBD = before bronchodilator use; postBD = after bronchodilator use; COPD is a postBD FEV_1_/FVC<lower limit of normal according to the PLATINO reference values.

*T test for independent groups or Fisher exact test for categorical variables; %P = FEV_1_ expressed as percentage of predicted by the PLATINO reference values; %B = FEV_1_ expressed as percentage of baseline or first measurement; Results based on 2,120 individuals with two preBD spirometric tests, or 2,026 individuals with two postBD spirometry tests.

The most consistent predictors of >100mL/y decline were the size of baseline function, age, response to bronchodilators, and in men smoking at baseline. Fifth percentiles of decline are also depicted in Table 2, and these were considerably higher (more negative) in men than in women, and higher than -40 mL/y. Table 2 includes the 5th percentile of the whole cohort, and the 5th percentile of a "respiratory healthy" group[15], comprised of never smoker individuals, lacking respiratory symptoms and previous diagnosis of respiratory diseases, demonstrating slightly more negative values than in the whole cohort.

Only about 6–11% of the variability of decline in women and 2–6% in men was explained by the statistical models.

## Discussion

Annual lung-function decline, one of the mechanisms leading to COPD, was estimated for a population-based cohort in three cities of Latin America. Decline was heterogeneous as previously reported in patients with COPD[18] but did not differ significantly in obstructed and non-obstructed individuals. In women, the presence of chronic bronchitis and frequent respiratory exacerbations also was associated with faster decline. As found in other longitudinal studies PostBD lung function declined faster (in mL/y) in men than in women, in individuals recruited at older age, in those with higher lung function at baseline, in current smokers, and in those with significant response to bronchodilators. A higher decline in mL/y in men and in those with higher lung function suggests that the decline is proportional to lung size[16], and models fitted to estimate loss as %P, or as %B, reduce the association of decline with baseline function. Expressing declines in mL/y may be deceiving as these tend to decrease with airflow obstruction, old age, women, and in individuals with small size, but if expressed as proportional changes, the declines may, in fact, increase[16].

We also estimated 5th percentiles of decline in the general population as it may be important for COPD risk and prevention, in that it could comprise a marker of a statistically significant and uncommon decline in the population. Fortunately, 5th percentiles of declines expressed as %P are more consistent around 3%/y, and those expressed as %B around 4%, which can be used to advantage. In our study, all declines expressed as %P, or as z-scores were statistically significant (lower than zero) meaning that the cross-sectional age-coefficient obtained from the baseline spirometry in healthy individuals[15] differs from the longitudinal age-decline.

A reduced postBD FEV_1_/FVC (COPD) was not associated with excessive decline if models were adjusted by baseline function, emphasizing the relevance of FEV_1_, rather than that of the FEV_1_/FVC ratio as predictors of decline, but also of mortality[7].

Individuals with previous physician diagnosis of TB, were associated with airflow obstruction and reduced baseline function in the PLATINO study[19], but they did not have a steeper lung-function decline consistently (see Online Supplementary Material). Data of South African miners exposed to silica and tuberculosis (TB), indicated that lung-function decline was proportional to the number of TB episodes[20]. However this may be different in the absence of significant exposure to silica in general population.

Similarly, individuals reporting exposure to biomass smoke or to dust at work were not more likely to have an increased rate of lung-function decline. Recently, a heterogeneous lung-function decline in a cohort of individuals with biomass smoke-associated COPD was reported[21], similar to that observed in other COPD cohorts associated with smoking[22]. In fact, only a small proportion of patients demonstrated a measurable decline.

In our study using multivariate models, individuals with a prior Physician diagnosis of asthma had significantly lower baseline FEV_1_ preBD, and postBD (-172 mL and -156 mL, respectively), but FEV_1_ declined similarly than the remaining of the cohort, in contrast with a steeper lung-function decline observed previously in asthmatics if the disease initiates in infancy, or is poorly controlled, or if the individual smokes[23].

Adult airflow obstruction may begin early in life, through poor lung development (small lungs), but also, as proposed years ago, individuals with normal -sized lungs may exhibit accelerated lung function-decline and develop obstruction[2]. Contrary to the traditional view, accelerated FEV_1_ decline in individuals with COPD is uncommon[22] and, in advanced COPD stages, decline in mL/y is reduced[24], although if expressed in relative terms, decline may increase[16] reinforcing the presence of a decline proportional to lung size or function and the convenience of expressing it as %B or %P.

In our study, mean FEV_1_ decline in COPD fell within the observed ranges in cohorts of patients with COPD[24], but, in addition the rates did not differ significantly from those of individuals without airflow obstruction, a comparison unavailable in data proceeding from cohorts of patients.

There are study limitations that should be mentioned: follow-up includes only three of the original five cities, with only two spirometric evaluations, limiting the accurate assessment of the annual individual FEV_1_ decline and possibly the identification of “fast decliners”[25]. In addition, participants from Mexico, likely with a higher Amerindian contribution, had a lower prevalence of COPD, although rates of decline in a cohort of Mexican patients coincide with those of the current report [21].

It is noteworthy that lung-function decline is highly variable even after several spirometric measurements[18] and also, that relevant information on lung-function decline has been produced based only on two measurements[26,27]. Although we performed only two spirometric tests, both were carried out with the same equipment, both included postBD measurements, and the test quality was good[15], with a population-based sample size sufficient for finding statistically significant and clinically relevant associations and high rates of follow-up after 5–9 years. Differently from studies based on cohorts of patients with COPD, our statistical models compared not only individuals with airflow obstruction due to their smoking experience, but also with non-obstructed individuals from general population. We reported mostly postBD spirometric measurements, which were also less variable in our data than those obtained prior to BD, and which represented maximal lung function at the testing time.

Smoking is the principal recognized cause of COPD, was a relevant risk for lung-function loss at baseline, and for lung-function decline (Table 4). Another priority comprises the diagnosis and control of asthma, a known cause of airflow obstruction and a relevant risk for exacerbations and lung function loss, and with effective interventions such as the use of inhaled corticosteroids.

In conclusion, obstructed and non-obstructed individuals had a similar lung-function decline in a population-based study from three Latin-American cities. Women with chronic cough and phlegm or with frequent respiratory exacerbations declined faster in FEV_1_. Anti-tobacco campaigns are the most important public health measure to prevent COPD. In addition, lung function decline should take into account baseline lung function and age, and expressing it as %P or %baseline entertains advantages over its customary expression as mL/y.

## Supporting information

S1 Appendix Flowchart describing the participants in the first and final examination of PLATINO study, and lost to follow-up. (DOCX)

S1 Table Multivariate regression coefficients (with 95% confidence intervals) for associations with the post bronchodilator log-Forced Expiratory Volume at one-second (logFEV_1_, logmL) decline in the cohort. (DOCX)

S2 Table Multivariate regression coefficients (with 95% confidence intervals) for associations with the post bronchodilator Forced Expiratory Volume at one second (FEV_1_, %) decline in the cohort expressed as percentage of baseline. (DOCX)

S3 Table Multivariate regression coefficients (with 95% confidence intervals) for associations with the post bronchodilator Forced Expiratory Volume at one-second (zFEV_1_) decline in the cohort expressed as Z score. (DOCX)

S4 Table Multivariate regression coefficients (with 95% confidence intervals) for associations with the post bronchodilator Forced Expiratory Volume at one-second (FEV_1 /_height^3^) decline in the cohort. (DOCX)

S5 Table Mean annual decline of FEV_1_ (logFEV_1_, zFEV_1_, FEV_1_/Height^3^) in individuals with and without airflow obstruction. (DOCX)
