# Supplementary material for: Lung function decline in subjects with and without COPD in a population-based cohort in Latin-America
Source: PLoS One. 2017 May 4;12(5):e0177032. doi: 10.1371/journal.pone.0177032 (PMC5417635; doi:10.1371/journal.pone.0177032)
Supplement: S1 Appendix — (DOCX) [file pone.0177032.s001.docx]

**S1-APPENDIX: FLOWCHART DESCRIBING THE PARTICIPANTS IN THE FIRST AND FINAL EXAMINATION OF PLATINO AND LOSS DURING FOLLOW-UP**

Participants in PLATINO baseline study with spirometry testing (n=3021 )

Had only preBD testing at baseline (n= 35 )

Post-bronchodilator spirometry at follow-up
(n= 2051 )

Refused post-bronchodilator spirometry (n= 69 )

Pre-bronchodilator spirometry (n= 2120 )

Refused spirometry or contraindicated (n= 78 )

Participated in follow-up (n= 2198 )

Dead before the 2nd examination (n= 301 )

Post-bronchodilator spirometry in both evaluations
 (n= 2016 )

Lost to follow up, usually moved and not localized (n= 522 )
