# Supplementary material for: Lung function decline in subjects with and without COPD in a population-based cohort in Latin-America
Source: PLoS One. 2017 May 4;12(5):e0177032. doi: 10.1371/journal.pone.0177032 (PMC5417635; doi:10.1371/journal.pone.0177032)
Supplement: S1 Table — (DOCX) [file pone.0177032.s002.docx]

s1-Table - Multivariate regression coefficients (with 95% confidence intervals) for associations with the post bronchodilator log-Forced Expiratory Volume at one second (logFEV_1_, logmL) decline in the cohort.

|  | Women | | | Men | | |
| --- | --- | --- | --- | --- | --- | --- |
|  | Mean decline | 95%CI | | Mean decline | 95%CI | |
| FEV1 at baseline | -0.000004 | -0.000007 | -0.000002 | 0.000004 | 0.000002 | 0.000006 |
| Age | -0.0003 | -0.0004 | -0.0002 | -0.0001 | -0.0003 | 0.0000 |
| Cigarettes/day at baseline | -0.0004 | -0.0005 | -0.0002 |  |  |  |
| Height (cm) | 0.0002 | 0.0001 | 0.0004 |  |  |  |
| BMI (Kg/m2) | 0.0001 | 0.0000 | 0.0003 | 0.0006 | 0.0003 | 0.0009 |
| Asthma |  |  |  | -0.0046* | -0.0094 | 0.0002 |
| FEV1/FVC<LLN |  |  |  | 0.008 | 0.002 | 0.014 |
| >2 exacerbations last year | -0.0076 | -0.0125 | -0.0026 |  |  |  |
| Chronic cough and phlegm | -0.0060 | -0.0108 | -0.0013 | -0.007 | -0.013 | -0.001 |
| Response to bronchodilators | -0.0053 | -0.0088 | -0.0017 | -0.037 | -0.054 | -0.021 |

95%CI = 95% confidence interval of the mean. PreBD= pre bronchodilator test; posBD= post bronchodilator test; %P= expressed as percentage of predicted according to PLATINO reference values. Variability explained by the model (adjusted R2) was 9% in women, and 4.9% in men. Bronchodilator response is the increase in FVC or FEV_1_ of ≥12% and of ≥200mL. Chronic cough and phlegm was cough or phlegm on the majority of days for >3 months in a year for >2 consecutive years. *All variables included in the models had a P<0.15, but some of the variables in the table do not reach the statistical significance at P<0.05 (95%CI including zero). Models based on 2,120 individuals with two preBD spirometric tests, or 2,026 individuals with two postBD spirometry tests.
