# Supplementary material for: Lung function decline in subjects with and without COPD in a population-based cohort in Latin-America
Source: PLoS One. 2017 May 4;12(5):e0177032. doi: 10.1371/journal.pone.0177032 (PMC5417635; doi:10.1371/journal.pone.0177032)
Supplement: S2 Table — (DOCX) [file pone.0177032.s003.docx]

s2-Table - Multivariate regression coefficients (with 95% confidence intervals) for associations with the post bronchodilator Forced Expiratory Volume at one second (FEV_1_, %) decline in the cohort expressed as percentage of baseline.

|  | Women | | | Men | | |
| --- | --- | --- | --- | --- | --- | --- |
|  | Annual drop | 95%CI | | Annual drop | 95%CI | |
| FEV1 at baseline | -0.001* | -0.001 | 0.000 |  |  |  |
| Age | -0.027 | -0.037 | -0.016 | -0.017 | -0.028 | -0.007 |
| Cigarettes/day | -0.034 | -0.048 | -0.020 |  |  |  |
| Height (cm) | 0.027 | 0.012 | 0.043 | 0.018 | 0.002 | 0.033 |
| BMI (Kg/m2) | 0.012* | -0.002 | 0.026 | 0.050 | 0.025 | 0.076 |
| Asthma |  |  |  | -0.366* | -0.760 | 0.028 |
| FEV_1_/FVC<LLN |  |  |  | 0.480* | -0.028 | 0.987 |
| >2 exacerbations last year | -0.691 | -1.141 | -0.241 |  |  |  |
| Chronic cough and phlegm | -0.566 | -0.999 | -0.132 |  |  |  |
| Response to bronchodilators | -0.469 | -0.789 | -0.150 | -0.690 | -1.175 | -0.205 |

95%CI = 95% confidence interval of the mean. PreBD= pre bronchodilator test; posBD= post bronchodilator test; %P= expressed as percentage of predicted according to PLATINO reference values. Variability explained by the model (adjusted R2) was 9% in women, and 4.9% in men. Bronchodilator response is the increase in FVC or FEV_1_ of ≥12% and of ≥200mL. Chronic cough and phlegm was cough or phlegm on the majority of days for >3 months in a year for >2 consecutive years. *All variables included in the models had a P<0.15, but some of the variables in the table do not reach the statistical significance at P<0.05 (95%CI including zero). Models based on 2,120 individuals with two preBD spirometric tests, or 2,026 individuals with two postBD spirometry tests.
