# Supplementary material for: Lung function decline in subjects with and without COPD in a population-based cohort in Latin-America
Source: PLoS One. 2017 May 4;12(5):e0177032. doi: 10.1371/journal.pone.0177032 (PMC5417635; doi:10.1371/journal.pone.0177032)
Supplement: S3 Table — (DOCX) [file pone.0177032.s004.docx]

s3-Table- Multivariate regression coefficients (with 95% confidence intervals) for associations with the post bronchodilator Forced Expiratory Volume at one-second (zFEV_1_) decline in the cohort expressed as Z score.

|  | Coeff women | 95%CI |  | Coeff men | 95%CI |  |
| --- | --- | --- | --- | --- | --- | --- |
| FEV_1_ at baseline | -0.00008 | -0.00009 | -0.00006 | -0.00003 | -0.00004 | -0.00001 |
| Age | -0.001* | -0.002 | 0.000 | -0.001* | -0.002 | 0.000 |
| Cigarettes/day | -0.002 | -0.003 | -0.001 |  |  |  |
| Smokes at baseline |  |  |  | -0.023 | -0.037 | -0.009 |
| Height (cm) | 0.003 | 0.002 | 0.003 | 0.002 | 0.001 | 0.003 |
| BMI (Kg/m2) |  |  |  | 0.002 | 0.000 | 0.004 |
| TB | -0.036 | -0.064 | -0.008 |  |  |  |
| >2 exacerbations last year | -0.031 | -0.058 | -0.005 |  |  |  |
| Chronic cough and phlegm | -0.038 | -0.063 | -0.013 | 0.028* | -0.006 | 0.062 |
| Response to bronchodilators | -0.033 | -0.051 | -0.014 | -0.041 | -0.069 | -0.012 |

95%CI = 95% confidence interval of the mean. PreBD= pre bronchodilator test; posBD= post bronchodilator test; %P= expressed as percentage of predicted according to PLATINO reference values. Variability explained by the model (adjusted R2) was 9% in women, and 4.9% in men. Bronchodilator response is the increase in FVC or FEV_1_ of ≥12% and of ≥200mL. Chronic cough and phlegm was cough or phlegm on the majority of days for >3 months in a year for >2 consecutive years. *All variables included in the models had a P<0.15, but some of the variables in the table do not reach the statistical significance at P<0.05 (95%CI including zero). Models based on 2,120 individuals with two preBD spirometric tests, or 2,026 individuals with two postBD spirometry tests.
