# Supplementary material for: Lung function decline in subjects with and without COPD in a population-based cohort in Latin-America
Source: PLoS One. 2017 May 4;12(5):e0177032. doi: 10.1371/journal.pone.0177032 (PMC5417635; doi:10.1371/journal.pone.0177032)
Supplement: S4 Table — (DOCX) [file pone.0177032.s005.docx]

s4-Table- Multivariate regression coefficients (with 95% confidence intervals) for associations with the post bronchodilator Forced Expiratory Volume at one-second (FEV_1 /_height^3^) decline in the cohort.

|  | Coef. | 95%CI |  | Men | 95%CI |  |
| --- | --- | --- | --- | --- | --- | --- |
| FEV_1_ at baseline | -0.008 | -0.009 | -0.006 | -0.003 | -0.004 | -0.001 |
| Age | -0.078 | -0.143 | -0.013 | -0.103 | -0.187 | -0.018 |
| Cigarettes/day | -0.195 | -0.282 | -0.107 |  |  |  |
| Smokes at baseline |  |  |  | -2.372 | -3.962 | -0.783 |
| Height (cm) | 0.474 | 0.377 | 0.571 | 0.341 | 0.222 | 0.459 |
| BMI (Kg/m2) | -0.065* | -0.154 | 0.024 | 0.212 | 0.037 | 0.386 |
| TB | -4.136 | -7.152 | -1.120 |  |  |  |
| >2 exacerbations last year | -2.800 | -5.589 | -0.011 |  |  |  |
| Chronic cough and phlegm | -4.492 | -7.179 | -1.805 |  |  |  |
| Response to bronchodilators | -3.409 | -5.390 | -1.428 | -4.536 | -7.750 | -1.322 |

95%CI = 95% confidence interval of the mean. PreBD= pre bronchodilator test; posBD= post bronchodilator test; %P= expressed as percentage of predicted according to PLATINO reference values. Variability explained by the model (adjusted R2) was 9% in women, and 4.9% in men. Bronchodilator response is the increase in FVC or FEV_1_ of ≥12% and of ≥200mL. Chronic cough and phlegm was cough or phlegm on the majority of days for >3 months in a year for >2 consecutive years. *All variables included in the models had a P<0.15, but some of the variables in the table do not reach the statistical significance at P<0.05 (95%CI including zero). Models based on 2,120 individuals with two preBD spirometric tests, or 2,026 individuals with two postBD spirometry tests.
