# Supplementary material for: Lung function decline in subjects with and without COPD in a population-based cohort in Latin-America
Source: PLoS One. 2017 May 4;12(5):e0177032. doi: 10.1371/journal.pone.0177032 (PMC5417635; doi:10.1371/journal.pone.0177032)
Supplement: S5 Table — (DOCX) [file pone.0177032.s006.docx]

s5-Table: mean annual decline of FEV_1_ (logFEV_1_, zFEV_1_, FEV_1_/Height^3^) in individuals with and without airflow obstruction

|  | NO COPD (1907)  Mean | SD (or 95%CI) | COPD (119)  Mean | SD or 95%CI | P* |
| --- | --- | --- | --- | --- | --- |
| Mean decline PreBD (log mL/y) | -0.015 | 0.021 | -0.016 | 0.03 | 0.5 |
| Mean decline PosBD (log mL/y) | -0.015 | 0.020 | -0.016 | 0.02 | 0.6 |
| Mean decline PreBD ZFEV_1_ | -0.011 | 0.10 | 0.006 | 0.11 | 0.10 |
| Mean decline PosBD ZFEV_1_ | -0.015 | 0.10 | 0.004 | 0.10 | 0.05 |
| Mean decline PreBD FEV_1_/Height^3^ | -7.4 | 11.6 | -6.4 | 11.5 | 0.57 |
| Mean decline PosBD FEV_1_/Height^3^ | -7.8 | 10.8 | -6.7 | 10.9 | 0.51 |

PreBD= pre bronchodilator test; posBD= post bronchodilator test; Airflow obstruction is a postBD FEV_1_/FVC<LLN.

*P values obtained by a T test for independent groups. 95%CI = 95% confidence interval of the mean. %P= expressed as percentage of predicted according to PLATINO reference values. zFEV_1_= FEV1 expressed as Z score according to the PLATINO reference values. Individuals compared were only those with 2 preBD or postBD spirometric tests. Results based on 2,120 individuals with two preBD spirometric tests, or 2,026 individuals with two postBD spirometry tests.
